# Application of hand-sewn esophagojejunostomy in laparoscopic total gastrectomy

**DOI:** 10.1186/s12957-024-03350-4

**Published:** 2024-03-02

**Authors:** Hao Gu, Weixiang Li, Lianbang Zhou

**Affiliations:** Department of General Surgery, the Second Affiliated Hospital of Anhui Medical University, Hefei, Anhui China

**Keywords:** Laparoscopic total gastrectomy, Digestive tract reconstruction, Hand-sewn esophagojejunostomy

## Abstract

**Objective:**

To investigate the clinical efficacy and prognostic implication of hand-sewn anastomosis in laparoscopic total gastrectomy (LTG).

**Methods:**

Retrospective analysis is adopted to the clinicopathologic data of 112 patients with gastric cancer (GC) who went through LTG in the Department of General Surgery, the Second Affiliated Hospital of Anhui Medical University between October 2020 and October 2022. Among them, 60 individuals receiving medical care were split into the hand-sewn anastomosis group (Group H, *N* = 60); while, 52 individuals were split into the circular stapler anastomosis group (Group C, *N* = 52) The clinical efficacy and prognostic conditions of hand-sewn anastomosis are compared with those of circular stapler anastomosis in the application of LTG.

**Results:**

The analysis results indicated that no notable difference was observed in intraoperative bleeding volume, time to first flatus (TFF), postoperative hospitalization duration and postoperative complications among the two groups (*P* > 0.05). Group H had shorter esophagojejunal anastomosis duration (20.0 min vs. 35.0 min) and surgery duration (252.6 ± 19.4 min vs. 265.9 ± 19.8 min), smaller incisions (5.0 cm vs. 10.5 cm), and lower hospitalization costs (58415.0 CNY vs. 63382.5 CNY) compared to Group C (*P* < 0.05).

**Conclusion:**

The clinical efficacy and the postoperative complications of hand-sewn esophagojejunostomy are basically equivalent in comparison to the circular stapler anastomosis in the application of LTG. Its advantage lies in shorter esophagojejunal anastomosis duration, shorter surgery duration, smaller incisions, lower hospitalization costs and wider adaptability of the location of the tumor.

## Background

Patients newly diagnosed with GC in China account for 40% of those worldwide every year. The morbidity and mortality of GC in China rank second and third, respectively, in the world [1]. Radical total gastrectomy (RTG) is the main therapeutic method for Siewert III and partial Siewert II adenocarcinomas of the esophagogastric junction (AEG) [2]. With the continuous maturity and promotion of laparoscopic technology, laparoscopic radical total gastrectomy (LRTG) has been routinely performed in many healthcare institutions with rich experience [3–5]. However, this surgical procedure still presents numerous technical challenges. Specifically, digestive tract reconstruction is an important link during GC surgery and also one of the difficult problems that persecute surgeons specializing in endoscopic GC surgery. Among the digestive tract reconstruction methods of LRTG, Roux-en-Y reconstruction is generally accepted as an ideal method at the present stage [6–8]. Currently, the most common device-assisted anastomosis in clinical practice includes linear anastomosis represented by overlap anastomosis and circular stapler anastomosis *via* auxiliary incisions. In overlap anastomosis, the anvil and cartridge of a linear stapler can be conveniently and safely placed into esophageal and jejunal lumens. Besides, since the anastomotic width is not limited by the thickness of esophageal and jejunal tubes, it is less likely to cause strictures. However, due to the side-to-side anastomosis procedure in overlap anastomosis, the severed end of the esophagus is dissociated with enough length to place the stapler cartridge for anastomosis. If the tumor invades the esophagus at a high position, the transabdominal anastomosis may become quite difficult. While the dissociation of the long lower esophagus is not required for the circular stapler anastomosis *via* auxiliary incisions. It can be explained that there is no reverse peristalsis as the circular stapler anastomosis is less limited by the position of tumor invasion and the anastomotic tension is not large, which contributes to food emptying. Nevertheless, there are also some obvious shortcomings for circular staplers. Specifically, an auxiliary small incision is necessary during the operation so that the stapler body can enter the abdominal cavity. After entering, the stapler body itself and the lifted intestinal loop will block the visual field and affect the process of anastomosis. Compared with device-assisted anastomosis, the procedure of hand-sewn anastomosis can be completed under direct vision. A favorable surgical field of vision exhibits prominent advantages for obese patients. The in-situ operation avoids tissue damage caused by excessive traction. However, performing hand-sewn anastomosis using a laparoscope is a complex task, so skilled suture technology is required. At present, hand-sewn esophagojejunostomy is only adopted in a few healthcare institutions [9–14]. Although there are many digestive tract reconstruction patterns during LTG, it remains undefined about the optimal anastomosis method [15, 16]. Hence, this research intended to assess and contrast the surgical efficacy and prognostic conditions between circular stapler anastomosis and hand-sewn anastomosis in LTG, thereby demonstrating the feasibility and superiority of hand-sewn anastomosis.

## Data and methods

### Patients screening

The retrospective collection of clinical data of 112 patients (Group H, *N* = 60; Group C, *N* = 52) with GC was adopted in this study who underwent LTG in the Department of General Surgery, the Second Affiliated Hospital of Anhui Medical University between October 2020 and October 2022. An aggregate of 112 patients experienced LTG + D2 lymphadenectomy [17] + Roux-en-Y anastomosis, all conducted by the same surgical team. Inclusion Criteria: (1) patients receiving preoperative electronic gastroscopy and histopathological examination and with a confirmed diagnosis of upper middle GC; (2) patients without distant metastases located in liver, bone or ovary by preoperative CT or MRI examination; (3) patients undergoing LTG; (4) patients with R0 resection verified by postoperative histopathological examinations; (5) patients with comprehensive clinicopathological information. Exclusion Criteria: (1) patients with gastrectomy for residual stomach; (2) patients with neoadjuvant chemoradiotherapy; (3) patients without D2 radical surgery; (4) patients with conversion to laparotomy; (5) patients with missing clinical pathological data.

### Surgical procedures

**Fig. 1:**
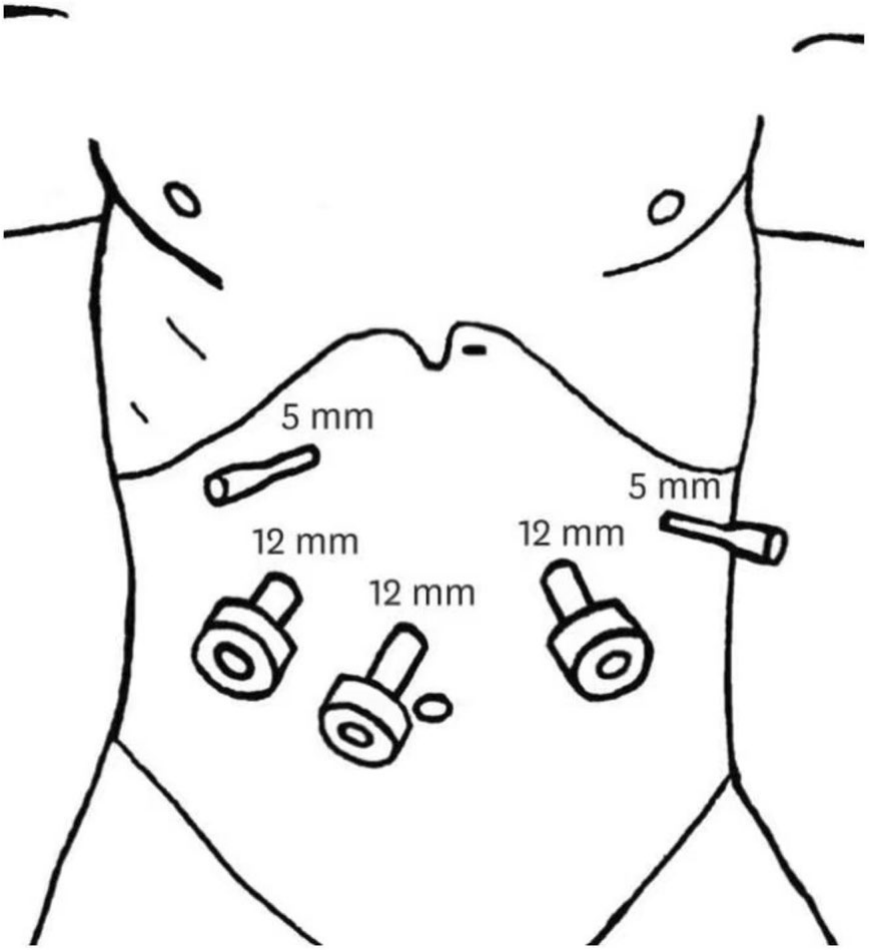
Establishing pneumoperitoneum

The pneumoperitoneum was routinely established for both patients of Group H and Group C (Fig. 1). The surgeon evaluates the location, size, and mobility of tumors by exploring the abdominal and pelvic cavity. Total gastrectomy and D2 lymphadenectomy were completed under direct vision by using a laparoscope.

#### Group H

**Fig. 2:**
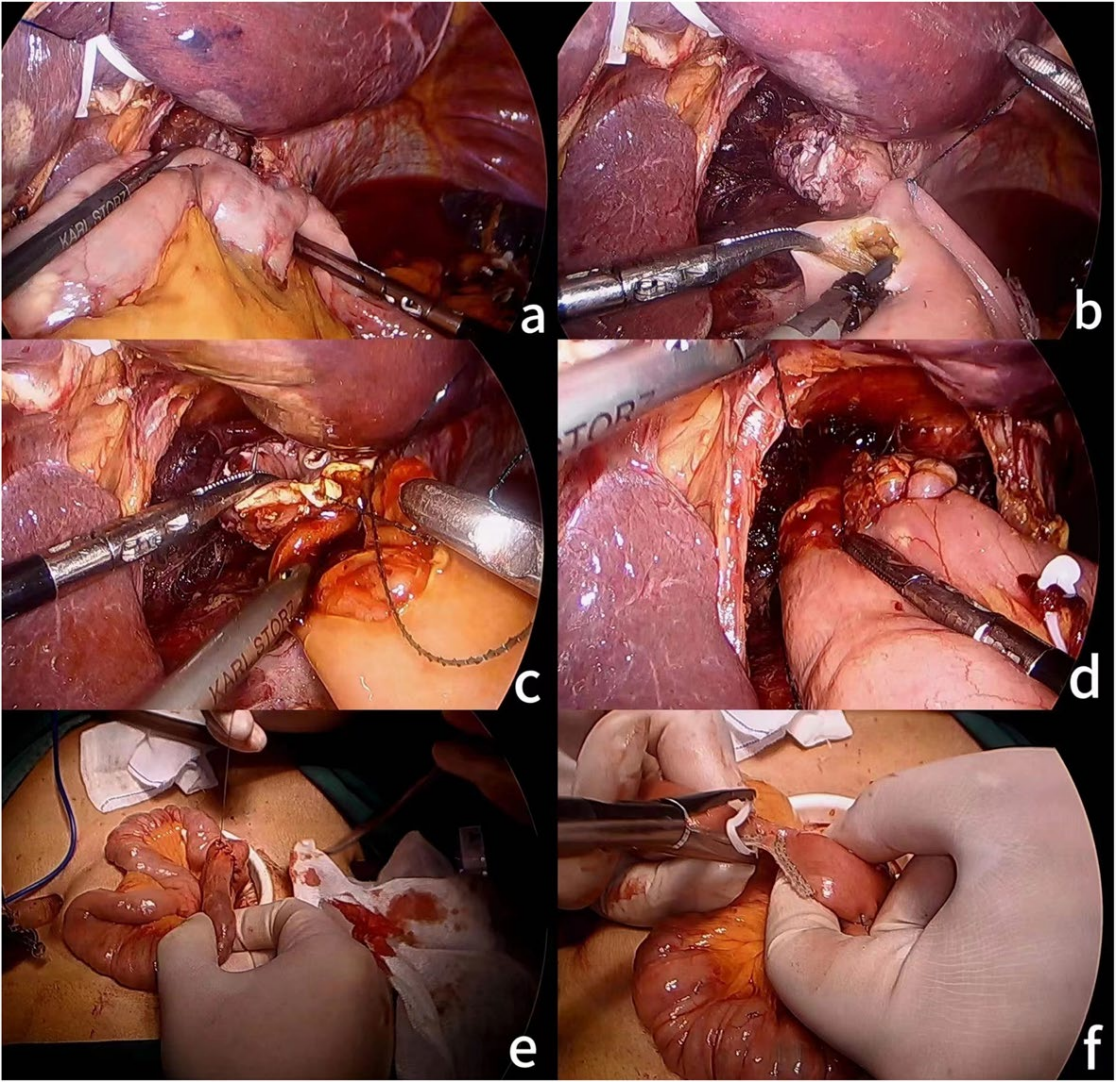
**a** check the tension of the raised jejunal intestinal canal; **b** make a 1.5 cm incision on the opposite mesenteric side wall of the jejunum; **c** manually suture the posterior wall of esophagojejunal anastomosis; **d** manually suture the anterior wall of esophagojejunal anastomosis; **e** manually suture common opening of side-to-side anastomosis of jejunum; **f** closed input the looped jejunum

**Fig. 3:**
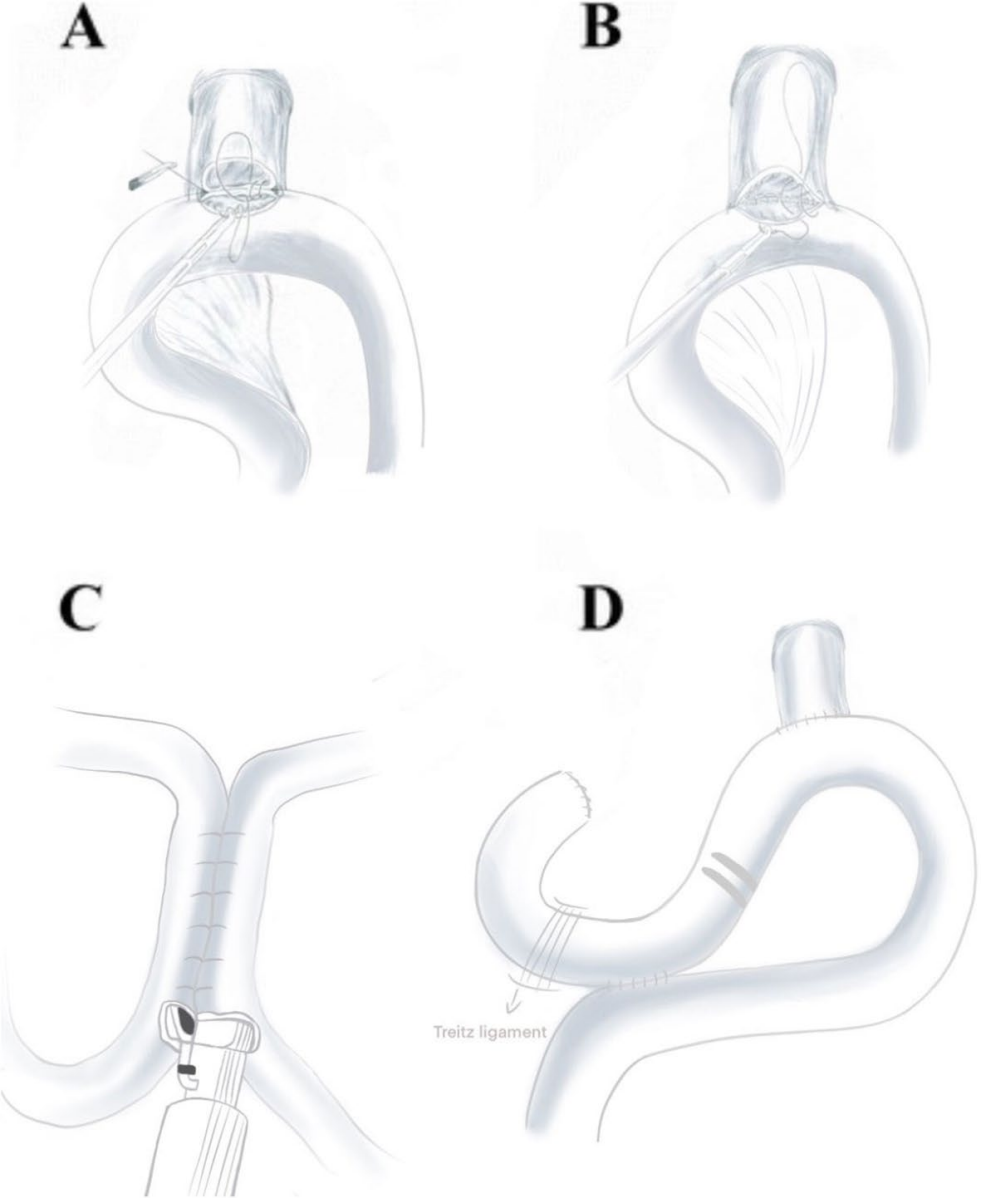
Process of hand-sewn esophagojejunostomy

The jejunum 25 cm away from the Treitz ligament was raised from the front of the transverse colon to the lower mediastinum, and the tension of the intestinal canal was checked. An ultrasonic scalpel is employed to create a 1.5 cm incision on the opposing mesenteric side wall of the jejunum, situated approximately 25 cm away from the Treitz ligament. A 3 − 0 barbed suture was utilized to perform full-thickness suturing of the posterior wall of the esophagojejunal anastomosis. This involved the placement of 6–8 stitches, proceeding from left to right in succession. A jejunal nutrition tube and gastric tube were placed about 25 cm below the esophagojejunal anastomosis. And then use the same method to suture the anterior wall of the esophagojejunal anastomosis. The two sutures were secured at the tail junction of the line, finalizing the end-to-side anastomosis between the esophagus and jejunum. The absorbable suture was utilized to fix the diaphragm and the anterior wall of the distal jejunum with 2 stitches to reduce the anastomotic tension. An ultrasound knife was used to make 0.5 cm incisions on the mesenteric side of the jejunum at the proximal end of the esophagojejunal anastomosis about 7-8 cm and the distal end about 40 cm, respectively. A linear cutting and closing device was employed to finalize the side-to-side anastomosis of the jejunum. Subsequently, a 3 − 0 barbed thread was utilized to suture the joint opening. Finally, the input loop jejunum 5 cm away from the proximal end of the esophagojejunal anastomosis was closed with an uncut linear cutting closure. As shown in Figs. 2 and 3.

#### Group C

An incision approximately 10 centimeters in length was created in the upper abdominal area, followed by the insertion of a sleeve to protect the incision. The free stomach and omentum were pulled out of the incision to expose the duodenum, and a linear cutting and closing device was used to sever the duodenum about 2 cm below the pylorus. The stomach body was pulled to expose the lower portion of the esophagus, and the esophagus was clamped about 2 cm above the toothed line with pouch forceps. The pouch needle suture was inserted, the distal esophagus was severed and the samples was extracted. Then the anvil holder was inserted into the pouch and the pouch line was knotted and fixed. The jejunum was cut approximately 20 cm from the Treitz ligament using a linear cutting closure device. The central rod of the circular stapler was extended from the opposing mesenteric side wall of the jejunum, situated about 5 cm away from the distal jejunum. The esophagojejunal end-to-side anastomosis was then performed in front of the transverse colon after docking the anvil base in the esophagus. The cut end of the jejunum was closed utilizing a linear cutting closure device. An ultrasonic knife was employed to create a 0.5 cm incision at approximately 15 cm from the Treitz ligament and on the mesenteric side wall of the jejunum, located about 40 cm away from the distal end. Subsequently, a linear cutting and closing device was utilized to complete the side-to-side anastomosis of the jejunum. The common opening was sutured with absorbable thread.

### Data collection and analysis

The surgical treatment (surgery duration, anastomosis duration, bleeding volume, and incision length), time to first flatus (TFF), postoperative hospitalization duration, hospitalization costs and complications within 6 months after surgery (anastomotic fistula, anastomotic stenosis, anastomotic hemorrhage, duodenal stump leakage, lymphatic leakage, pleural effusion, abdominal infection, incision infection, and intestinal obstruction…) were examined and compared in both groups. The patients were subsequently monitored 1, 3 and 6 months after surgery by telephone, outpatient review and inpatient examination, and the main follow-up items included whole-abdominal and chest CT, gastroscopy, upper gastrointestinal contrast, etc. The follow-up was up to April 2023.

SPSS 27.0 statistical software was adopted to perform data processing. For normally distributed measurement information, they were presented as mean ± standard deviation (x ± s), and the independent sample t-test was employed for group comparison. Qualitative data were presented as percentages (%), and the χ2 test was employed to compare between groups. A *P*-value of less than 0.05 was deemed to indicate statistical significance.

## Results

No significant differences were observed between the two groups in terms of gender, age, the BMI (Body Mass Index), tumor diameter length, tumor location, TNM stage and ASA (American Society of Anesthesiologists) grades (*P* > 0.05), as presented in Table 1. No significant differences were observed in the volume of intraoperative bleeding, time to first flatus (TFF), duration of postoperative hospitalization, and postoperative complications between the two groups (*P* > 0.05); Group H had shorter esophagojejunal anastomosis duration (20.0 min vs. 35.0 min) and surgery duration (252.6 ± 19.4 min vs. 265.9 ± 19.8 min ), smaller incisions (5.0 cm vs. 10.5 cm), and lower hospitalization costs (58415.0 CNY vs. 63382.5 CNY) in comparison to Group C (*P* < 0.05), as illustrated in Tables 2 and 3.

**Table 1:** Comparison of general data between group H and group C

Group	Cases	Gender [cases (%)]			Age [*M*(*P*_25_, *P*_75_), years]	BMI ($\mathrm{\overline x}$±s, kg/m^2^)	Tumor location [cases (%)]	
		Male	Female				Cardia	Stomach body
H	60	44(73.3)	16(26.7)		69.0(63.0, 72.0)	22.1±2.4	42(70.0)	18(30.0)
C	52	37(71.2)	15(28.8)		68.0(61.3, 74.8)	22.7±1.7	40(76.9)	12(23.1)
Statistical value		*χ*^*2*^=0.066			*Z*= -0.184	*t*= -1.413	*χ*^*2*^=0.681	
*P value*		0.797			0.854	0.160	0.409	
Group	Cases	TNM staging [cases (%)]			Tumor length ($\mathrm{\overline x}$±s, cm)	ASA classification [cases (%)]		
		I	II	III		I	II	III
H	60	15(25.0)	22(36.7)	23(38.3)	4.2±1.60	17(28.3)	37(61.7)	6(10.0)
C	52	7(13.5)	33(63.4)	12(23.1)	4.0±1.50	15(28.8)	29(55.8)	8(15.4)
Statistical value		*Z*=-0.429			*t*=0.581	*Z*=-0.352		
*P value *		0.668			0.563	0.725		

**Table 2:** Comparison of intraoperative and postoperative conditions between group H and group C

Item	Group H (*n* = 60)	Group C (*n* = 52)	Statistical value	* P* value
Surgery duration ($\mathrm{\overline x}$± s, min)	252.6 ± 19.4	265.9 ± 19.8	*t*=-3.581	< 0.001
Esophagojejunal anastomosis duration[*M*(*P*_25_, *P*_75_), *min*]	20.0(19.0, 22.0)	35.0(33.25, 36.0)	*Z*=-9.130	< 0.001
Incision length [*M*(*P*_25_, *P*_75_), *cm*]	5.0(5.0, 5.0)	10.5(10.0, 11.0)	*Z*=-9.358	< 0.001
Intraoperative bleeding volume ($\mathrm{\overline x}$ ± s, ml)	75.0(65.0, 95.0)	77.5(70.0, 85.0)	*Z*=-0.123	0.902
time to first flatus [*M*(*P*_25_, *P*_75_), *d*]	3.0(3.0, 4.0)	3.0(3.0, 4.0)	*Z*=-0.098	0.922
Postoperative hospitalization duration [*M*(*P*_25_, *P*_75_), *d*]	9.0(9.0, 10.75)	9.0(9.0, 10.75)	*Z*=-0.264	0.792
Hospitalization costs [*M*(*P*_25_, *P*_75_), CNY ]	58415.0(55779.29, 62505.25)	63382.5(61279.75, 65749.25)	*Z*=-4.271	< 0.001

**Table 3:** Comparison of postoperative complications between group H and group C

Item	Group H (*n* = 60)	Group C (*n* = 52)	Statistical value	* P* value
Postoperative complications [cases (%)]				
Total cases	18(30.0)	21(40.4)	*χ*^*2*^ = 1.324	0.250
Esophagojejunal anastomotic fistula	1(1.7)	2(3.8)	*χ*^*2*^ = 0.508	0.476
Esophagojejunal anastomotic stenosis	1(1.7)	3(5.8)	*χ*^*2*^ = 1.361	0.243
Esophagojejunal anastomotic hemorrhage	0	2(3.8)	*χ*^*2*^ = 2.350	0.125
Duodenal stump leakage	2(3.3)	1(1.9)	*χ*^*2*^ = 0.213	0.645
Lymphatic leakage	4(6.7)	3(5.8)	*χ*^*2*^ = 0.038	0.845
Pleural effusion	2(3.3)	2(3.8)	*χ*^*2*^ = 0.021	0.884
Reflux esophagitis	3(5.0)	2(3.8)	*χ*^*2*^ = 0.087	0.768
Incisional infection	2(3.3)	4(7.7)	*χ*^*2*^ = 1.044	0.307
Intestinal obstruction	3(5.0)	2(3.8)	*χ*^*2*^ = 0.087	0.768
Clavien-Dindo classification [cases (%)]				
I and II	13(21.7)	15(28.8)	*χ*^*2*^ = 0.766	0.382
≥III	5(8.3)	6(11.5)	*χ*^*2*^ = 0.323	0.570

## Discussion

In the study, the anastomosis duration and operation duration of group C were longer than those of group H, which was the same as reported by Honório et al. [18]. We believe that there are technical difficulties in group C, such as the placement of the anvil holder and pouch suture, and the need to make a small incision to pass the Trocar. The operation steps are cumbersome, so it takes a long time. In group H, skilled suturing techniques enabled the direct and continuous full-thickness suturing of both the anterior and posterior walls of the esophagus and jejunum, significantly reducing the time required for digestive tract reconstruction. In addition, we used barbed thread for hand stitching during the stitching process. The self-fixation characteristic of barbed thread can effectively prevent tissue sliding in a continuous suture, which shortens the suture time to a certain extent [19–21]. Carter et al. [22] believed that longer operation time might affect the prognosis of patients. In this study, however, the prognosis between the two groups did not demonstrate a notable distinction (*P* > 0.05). Both circular anastomosis techniques through the auxiliary incision and hand-sewn anastomosis techniques have been carried out in our center for many years, and the surgical team has a solid operational foundation, so the prognosis of the two groups of patients is similar.

According to research data [23], the risk of anastomotic stenosis after circular stapling was about 2.8%. In this study, the occurrence of anastomotic stenosis was1.7% (1/60) in Group H and 5.8% (3/52) in Group C. We believe that the circular anastomosis is perpendicular to the esophageal lumen and prone to cicatricial stenosis. Hand-sewn anastomosis does not require the insertion of a stapler, which can reduce the damage of the anastomosis. Simultaneously, we employ an absorbable suture for anastomosis closure. This approach facilitates easy expansion in cases where the anastomosis is narrow. There was one patient with postoperative anastomotic stenosis in Group H. The patient underwent anastomotic dilation under gastroscopy after hospitalization, and the symptoms of feeding difficulties were immediately relieved.

Regarding anastomotic fistula, a significant postoperative complication, this study found incidences of 1.7% (1/60) in Group H and 3.8% (2/52) in Group C. In comparison, the study by Inokuchi M et al. [24] reported a 3.0% incidence of anastomotic fistula in LTG. We think that hand-sewn anastomosis has certain advantages in this respect: the laparoscopic field of view enables the clear and precise execution of hand-sewn anastomosis; In addition, the jejunum and its mesangium were not severed in the process of end-to-side esophagojejunal anastomosis, and the anastomosis had better blood flow, which decreased the occurrence of anastomotic fistula.

Reviewing the surgical data of this study, we found that 2 patients were evaluated as Siewert type III AEG before surgery. However, the tumor was found to have involved the dentate line during surgery. And the lower 3 cm esophagus had to be removed. While this approach raises the complexity of hand-sewn anastomosis, it also demonstrates that, given proficient suturing skills, hand-sewn anastomosis offers greater adaptability to varying tumor locations. For patients with positive incisal margin confirmed by intraoperative frozen section, we can extend the length of esophagectomy appropriately to confirm negative incisal margin before performing the anastomosis. Consequently, hand-sewn anastomosis has the potential to enhance the R0 resection rate and, to a certain extent, decrease the rate of conversion to laparotomy [25].

Compared with device-assisted anastomosis, hand-sewn anastomosis possesses a lower surgical cost (*P* < 0.05) due to the high cost of the stapler device [18]. When the surgical outcomes and postoperative complications of Group H and Group C are similar, hand-sewn anastomosis stands as a secure and economically viable alternative.

### Limitations

Initially, the constraints of this study encompass its retrospective design and a somewhat limited sample size. Secondly, the short follow-up period might have contributed to the lack of statistical difference observed in the long-term quality of life. Finally, circular stapler anastomosis includes the Orvil method, reverse puncture method, and purse suture method. While in this study, the purse suture method is used for all circular stapler anastomosis. In other words, only one circular stapler anastomosis method is compared with hand-sewn anastomosis, which may induce some selection bias.

## Conclusion

The clinical effectiveness and postoperative complications of hand-sewn esophagojejunostomy are essentially on par with those of circular stapler anastomosis when applied in Laparoscopic Total Gastrectomy (LTG). Its advantage lies in shorter esophagojejunal anastomosis duration, shorter surgery duration, smaller incisions, lower hospitalization costs and wider adaptability of the location of the tumor.

## Data Availability

No datasets were generated or analysed during the current study.

## Abbreviations

LTG: Laparoscopic total gastrectomy
GC: Gastric Cancer
TFF: Time to first flatus
RTG: Radical total gastrectomy
AEG: Adenocarcinoma of the esophagogastric junction
LRTG: Laparoscopic radical total gastrectomy
PSS: purse-string suture
ASA: American Society of Anesthesiologists
BMI: Body mass index
